# The Relevance of the Procedures Related to the Physiotherapy in the Interventions in Patients with Prostate Cancer: Short Review with Practice Approach

**Published:** 2014-06

**Authors:** Mario Bernardo-Filho, Mauro Luis Barbosa Júnior, Danúbia da Cunha Sá-Caputo, Eliane de Oliveira Guedes de Aguiar, Rafaelle Pacheco Carvalho de Lima, Sebastião David Santos-Filho, Severo de Paoli, Giuseppe Antonio Presta, Milena de Oliveira Bravo Monteiro, Ângela Tavares

**Affiliations:** 1 Departamento de Biofísica e Biometria, Instituto de Biologia Roberto Alcântara Gomes, Universidade do Estado do Rio de Janeiro, 20551-030, Rio de Janeiro, RJ, Brasil;; 2 Departamento de Medicina Integral Familiar e Comunitária, Hospital Universitário Pedro Ernesto, Universidade do Estado do Rio de Janeiro, 20551-030, Rio de Janeiro, RJ, Brasil;; 3 Mestrado Profissional em Saúde, Medicina Laboratorial e Tecnologia Forense, Universidade do Estado do Rio de Janeiro, 20551-030, Rio de Janeiro, RJ, Brasil;; 4 Departamento de Morfologia, Centro de Ciências da Saúde, Universidade Estácio de Sá, 20261-060, Rio de Janeiro, RJ, Brasil;; 5 Departamento de Ciências Fisiológicas, Instituto de Ciências Biológicas e da Saúde, Universidade Federal do Estado do Rio de Janeiro, Rio de Janeiro, RJ, Brasil;; 6 Programa de Pós-Graduação em Ciências Médicas, Instituto de Biologia Roberto Alcântara Gomes, Universidade do Estado do Rio de Janeiro, 20551-030, Rio de Janeiro, RJ, Brasil;; 7 Faculdade Redentor, Campus Itaperuna, 28300-000, Itaperuna, RJ, Brasil

**Keywords:** prostate cancer, physiotherapy, urinary incontinence, erectile dysfunction

## Abstract

Advances in medical science procedures and their utilization in the field of oncology improved the survival of patients. In consequence, these advances have influenced the practice of physiotherapy. Physiotherapists utilize physical agents with the objective to enhance the health, welfare and quality of life and thus they can play important role in the management and rehabilitation of patients with prostate cancer (PCa). Urinary incontinence (UI) and erectile dysfunction (ED) are effects normally associated with the radical prostatectomy and radiotherapy due to the damage of the muscles of the pelvic floor (MPV). The aim of this work is to present findings related to the PCa and how the physiotherapist can guide the patient in relation to the knowledge and understanding of the anatomic structures related directly with the pelvic floor, the correct breathing and the perception of the MPV, as other muscles of the pelvis. Interventions of the physiotherapy will re-train the muscles of the pelvis by improving the active retention strength of the MPV in order to overcome the insufficiency (mainly the UI and ED) of the injured muscles. In conclusion, it is suggested to consider and to offer to the PCa patients the techniques related to the physiotherapy before and after the treatment.

## INTRODUCTION

Cancer is characterized by an uncontrolled growth of abnormal cells in the body and it is considered a national health priority area in several countries due to the burden that it places on the individual, families and the community (1-3).

An important consideration is that the non-communicable diseases (NCD), including cancer, are overtaking infectious disease as the leading health-care threat in some of middle-income and low-income countries and NCD are the major health and socio-economic issue. In general, Latin American and Caribbean countries are struggling to try to respond to increasing morbidity and death from advanced disease. The health-care systems in these countries face many challenges to treat patients with advanced cancer, due to (i) inadequate funding; (ii) inequitable distribution of resources and services; (iii) training, and distribution of health-care personnel and equipment; (iv) lack of adequate care for many populations and (v) geographic situation pf the houses of the patients. Moreover, the information to the prevention is not totally available to the entire population (3, 4a, 4b).

Nevertheless, advances in health and in general biomedical investigations have improved survival and quality of life of the patients with cancer. In consequence, older cancer survivors have represented an important group in the population (4c-6). These considerations must be discussed and they have thus strongly influenced the practice of the physiotherapy (1-3; 4c-6).

Besides the knowledge about clinical interventions, the physiotherapist needs to be in contact with the recent advances, in general, of the scientific literature involving cancer. Moreover, this professional must know about the risk factors and participate in actions and attempts to aid in the prevention of this disease (7-9).

The risk of some cancers, such as prostate, may be reduced in people living in areas of high ambient solar radiation or with high sun exposure in comparison with the individuals where the converse is the case (10-12). In addition, Khadilkar and Khadilkar, 2012 (13) have reported that an inverse relationship has been found between sunlight exposure and rates of incidence of death for certain cancers in particular geographic areas based in the studies published by Boscoe and Schymura, 2006 (14). Evidence of a possible cancer-protective role for vitamin D has also been observed in laboratory studies of the effect of vitamin D treatment on cancer cells in culture.

Naturally, the information about the protection against the unnecessary and undesirable exposition of the sunlight is also very important (15, 16).

The general knowledge about the actions to aid to prevent the prostate cancer (PCa) is highly desirable, due to undesirable clinical conditions due to the consequences of some techniques used to treat the Pca, as the radical prostatectomy (RP) and the radiotherapy (RT). These conditions can bring bothersome, as the urinary incontinence (UI) and the erectile dysfunction (ED), as they can comprise directly the sexual health and the quality of life of the patient. It is important that the interprofessional team, including a physiotherapist, be prepared to discuss these questions and to present strategies to help the patient in the various stages of the treatment (17, 18).

### Approaches in cancer and the general role of the physiotherapy

The procedures of the physiotherapy, in general, have the purpose of developing, maintaining and restoring the maximum movement and functional ability of each individual, considering the specific limitations, the disease and the medications related to the patient. The role of the physiotherapist within the interdisciplinary group is well defined in various clinical conditions, as with the patient with PCa (7, 9, 19).

Concerning to the PCa, physiotherapeutic procedures are also relevant in the treatment, in the prevention of diseases or complications and in the management or treatment of undesirable pathological conditions to thus abolish or minimize the impact that these may have in the quality of life of the patient (8). Baumann *et al,* 2012 (20) have reported that UI, ED, fatigue as well as fears and depression rank are among the most common complaints in patients with PCa.

In general, physiotherapy management in the area of oncology have relevant contributions to patient care, including the actions indicated in Table 1, in which, some considerations are presented. Furthermore, they demonstrate how the disciplines allied to medicine are working together to bring the healthy individual back to normal life and re-integration to the Society, or improve the quality of life of patients that have to live with cancer as a chronic disorder and those that are in the terminal stages of the disease and life (7-9, 19, 21, 22).

**Table 1:** General managements of the physiotherapy in oncology

Action	Consideration

Decreasing length of stay in acute facilities	Early discharge planning, outpatient follow up and education, involvement in palliative care facilities and physiotherapy services in home care
Improving functional capacity	Early moblilization, management of complications of surgery, convenient manipulations of the areas submitted to RT and other treatment, as treating lymphoedema and scars
Improving lymphoedema management	Decreasing hospital admissions for cellulitis (a feature of poorly controlled lymphoedema and/or orientation of the patient) and decreased need for costly and at times uncomfortable pressure garments
Improving local and general exercise capacity	Prevention of loss of body weight and managing the side effects of the disease, medication and surgery
Shortening the period of time of UI after RP	Increasing of quality of the life of the patient
Improving quality of life factors	Increasing of quality of the life for all patients with cancer and their carers and families

RP, radical prostatectomy; RT, radiotherapy.

The pysiotherapist working in oncology must have a broad knowledge in neurology, in the musculoskeletal and cardiopulmonary systems and in rehabilitation, kinesiotherapy and electrotherapy to perform correctly the management of the patients, as well as in assisting people’s return to work and normal daily life (7, 17). It is desirable the knowledge of the consequences and complications of clinical procedures. A discussion about these procedures and the possible complications and occurrences are relevant to the management of the patient before and after the surgery (6, 8, 17, 23).

In addition, the physiotherapist also needs to know about the medications. Hormonal therapy, for example, has an important effect on the muscle mass. The decrease in muscle mass, leading to muscle weakness and general debility, can be minimized by specific kinesiotherapy programmes (6-8, 17). Involving the movements of the body and the optimization of the functions of the tissues, the kinesiotherapy aims to enhance the health, welfare and quality of life and thus they can play an important role in the management and rehabilitation of patients. Ness *et al,* 2006 (24) have reported that physical exercise during and after cancer treatment has been shown to be effective to reduce several negative clinical consequences followed by cancer and cancer treatment.

Considering the PCa patient, the physiotherapist will also guide in general approaches and in relation to (i) the knowledge and understanding of the anatomic structures related directly with the pelvic floor, (ii) the correct breathing and (iii) the perception of the muscles of the pelvic floor, as other muscles of the pelvis. Specific attention is given to the comprehension of the functions of these muscles, especially to the relevance of the levator ani muscle (23, 25-31). Moreover, Dorey *et al*, 2009 (23), during each therapy appointment with the PCa patient, pelvic floor muscle contraction strength has been assessed by a digital anal examination using the Oxford Score modified by Dorey, 2006 (29) that has included Grades 0 to 6. This new Score (29) has been utilized to evaluate the strength of the anal sphincter and puborectalis muscle in men. Verbal feedback from this examination has been considered to teach the men how to contract their muscles of the pelvic floor optimally. Furthermore, they are advised on improvement from previous appointments related to the examinations that were performed.

UI and ED has been also treated with the various exercises (kinesiotherapy) involving the muscles of the pelvic floor in patients submitted to RP. Prior to a pelvic floor muscle exercise program, an anal assessment is performed to grade the strength, endurance and speed of the anal sphincter and the puborectalis muscle. Pelvic floor muscle exercises are individually taught to ensure that they are being performed correctly (23, 32). The muscles of the pelvic floor have several functions, as the flatus, fecal and urinary continence. In addition, these muscles are also strongly involved with the sexual function (26, 30, 33).

Sexual health is a state of physical, emotional, mental and social well-being in relation to sexuality. Sexuality is influenced by the interaction of biological, psychological, social, economic, political, cultural, ethical, legal, historical, religious and spiritual factors (34-36). Considering the biological factors of the sexuality (37), the procedures used in the physiotherapy will be helpful and thhe physiotherapist must have also knowledge about the sexuality to define specific physical exercises and other techniques available to aid the patient with PCa in different steps of his life, as well as the limitations of these and other procedures (8, 18, 33).

**Figure 1:**
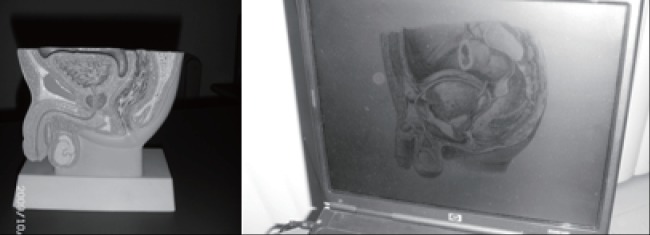
Tools used to explain to the patient about the anatomic structures of the pelvic floor

Figure 1 show some tools used to explain the patient with PCa about the anatomic structures directly and indirectly involved with the prostate and the structures that can be damaged in the surgery, as well as in the radiotherapy. The images in the computer are important, but the model of the structures in mass is worthwhile to the patient. Several authors have suggested the use of tools to allow the patient to understand and to learn better about the aim of the procedures that are involved with the treatment (23, 28, 30).

### Prostate cancer in the world

PCa is the most commonly diagnosed solid cancer among men worldwide and is the most frequent of all cancers in Europe and North America. Mortality rates tend to be higher in less developed regions of the world including parts of South America, the Caribbean, and sub-Saharan Africa (2, 6, 38, 39).

In Table 2 are shown the mainly risk factors for PCa. Furthermore, some considerations about these risk factors are also presented.

**Table 2:** Mainly risk factors for prostate cancer

Risk factor	Consideration

Age	It is the strongest risk factor
Family history	Greater risk if father or brother had the disease and slightly higher for men whose mothers or sisters have had breast cancer
Race/Ethnicity	Greater risk among African American men compared with white, Asian, and American India men
Prostate changes	Abnormal cells described as high-grade prostatic intraepithelial neoplasia
Diet/food	It is an important risk factor food with high animal fat and low in fruits and vegetables

The high relevance of the cancer can also be demonstrated by the number of scientific research (2,677,538 publications) identified on September 10th 2012 in the database system PubMed (a service of the National Library of Medicine and the National Institutes of Health) (40). The keyword “prostate cancer” yields 60,087 publications, that is 2.24% of the publications in cancer.

The early diagnosis of PCa has been facilitated by the determination of the prostate specific antigen (PSA), rectal touch and ultrasonography, which has subsequently led to a high cure rate in the early stages (stage I/II) of the disease. (2, 8, 41-43). When a man has the PCa early diagnosed, he has a number of treatment options, which carry similar success rates. Surgery, brachytherapy or external beam radiotherapy in combination with several months of initial hormone treatment all carry the same chance of cure but they all have very different recovery times, or number of visits to the hospital to consider (8, 44). In addition, with the early diagnosis there is an expectation of curing cancer, minimizing the risk of UI and ED and increasing the quality of life of the patient (45-48).

### Some procedures to treat the prostate cancer

In general, RP is a curative and appropriated therapy for any patient whose tumour is clinically confined to the prostate. Other factors affecting treatment decisions include patient factors, such as (i) Current symptoms (International Prostate Symptom Score, urinary flow rate), (ii) Current age (preference under the age of 70 years), (iii) Concurrent illnesses may determine suitability or not for surgery, (iv) Patient preference (psychological factors including patients ideas, concerns and expectations) (45-48).

In Table 3 are shown some tumor/cancer factors that must considered in the approaches related to the PCa that are relevant in the evolution and decisions in the clinical practice (8, 39, 44-48).

**Table 3:** Tumor/cancer factors that must considered in the approaches related to the PCa

Factors	Consideration

Grade of tumour	“Aggressiveness” determines the risk of relapse
Stage of tumour	It determines radical or palliative approach
Chance of response to treatment	Early diagnosis
Chance of recurrence	Delayed diagnosis
Possibility of second curative treatment modality	If the first treatment fails

RT is another option for treatment of PCa and it uses high-energy X-rays or other types of ionizing radiation to try to kill the cancer cells in various organs/tissues. In addition, RT combined with androgen deprivation therapy (ADT) is used in patients with locally advanced tumors and/or those with high Gleason scores, characterized as intermediate or high-risk profiles (49).

Table 4 shows the mainly two types of radiotherapy used in oncology and some biophysical considerations (8, 44).

**Table 4:** Types of radiotherapy used in oncology

Type of Radiotherapy	Denomination	Biophysical consideration

External radiotherapy	Teletherapy	It uses a source of ionizing radiation that is placed outside of the body.
Internal radiotherapy	Brachytherapy	It uses a radioactive source sealed in needles, seeds, wires, or catheters placed directly into or closed to the tumour.

### Undesirable findings related to the procedures used to treat prostate cancer

Naturally, the clinical conditions and the early diagnosis of the PCa will minimize the undesirable findings due to the therapeutic procedure. Severe complications following RT can occur and these undesirable findings depend on the type of the procedure used in the surgery and UI and ED have also been associated with the RT (8, 18, 47, 50).

As presented before, UI and ED are undesirable side effects normally associated with the RP and RT due to the damage of the muscles of the pelvic floor (8, 21, 30, 45, 46). UI has a prevalence ranging from 5 to 60%. In the clinical routine with the patient that was submitted to treatment to PCa, it is verified that the UI is an unpleasant condition (26, 29, 30). The impact of UI on the quality of life of the PCa patient is determined by the self-perception of the severity and the disruption of daily activities caused by the symptoms. An important consideration is that the cases of UI and ED (fecal incontinence and another sexual dysfunctions, as loss of ejaculation, decreased libid and orgasm) recorded in clinics seem to be much higher than the number described in the publications. This discrepancy could be attributed to the great variability of definitions, measurement instruments, and manners of assessing the clinical disorder (8, 25, 29, 50).

ED, in general, is usually due to a multifactorial etiology, comprising organic, psychological, or mixed aspects, and may often require a multidisciplinary approach for assessment and treatment. Organic causes encompass vascular, neurologic, hormonal, as a result of medications, pelvic surgery (mainly RP), RT, diabetes or mixed factors. In general, any condition that can cause damages to the nerves or impair blood flow in the penis may lead to ED. Pelvic surgery (especially RP and bladder surgery for cancer) might damage cavernous nerves and arteries near the penis, causing ED (23, 46).

The economic burden of ED is not just limited to the cost of diagnosis and treatment. Other impacts on the Society and in diary life that are difficult to quantify are shown in Table 5.

**Table 5:** Impacts of the erectile dysfunction on the individual and on the Society

Impact on the Sociely

Lost time at work
Decreased productivity of the patient due to distress
Negative Impact on the partner and family
Impairment of the social interactions

The comprehensive knowledge and the understanding of these impacts have also reflected in the publications in important scientific journals that have increased along of the years (34, 45, 46, 48). Reports of studies describing ED after RP have shown a range from 29% to 97.5% with less ED occurring in younger men. Men with ED may suffer from depression and low self-esteem, and experience difficulties establishing and maintaining relationships. In addition to ED, RP has been also associated with a 50% weaking of the orgasmic sensation (51). Treatment regimens currently available for ED include psychotherapy, sex therapy, oral pharmacological agents, androgen replacement therapy, intraurethral therapy, intracavernosal injections, several procedures related to the physiotherapy and surgery (23, 34, 45, 46, 48, 52). Dorey *et al*, 2009 (23) has reported that contracting the pelvic floor muscles during sexual activity has been also recommended to the patient to maintain or improve erectile strength.

### Physiotherapy procedures in the management of the patient with prostate cancer

The terms physiotherapy and physical therapy are associated with several techniques that are used in the treatment and in the rehabilitation of patients in various clinical status. The determination of the number of publications found in the PubMed using the keyword cancer and “physical therapy” yields 605 papers and, with physiotherapy 12 068 papers. The percentage of publications involving “physical therapy” and physiotherapy with cancer are 0.037% and 0.45%, respectively. If, it is considered the keyword “prostate cancer” and “physical therapy” is found 16 papers and, with physiotherapy 308 papers. The percentage of the number of publications involving “physical therapy” and physiotherapy with “prostate cancer” are 0.026% and 0.51%, respectively. It is possible to see that the interest in the publication of findings involving cancer or prostate cancer and physical therapy or physiotherapy is extremely low. Nevertheless, when it is used the keyword physiotherapy is possible to find a percentage 10 times bigger that with physical therapy. This finding is important and it can aid the professional of the Health Area to find additional information related to treatments involving cancer and the use of the keyword physiotherapy may be important. In the clinical practice is very important that the professional be in contact with the development of new treatment techniques. Researches into the causes, prevention, and treatment of various diseases are ongoing in many biomedical centres throughout the world. It is therefore vital that physicians, as well as, other professionals of the Health Area, as the physiotherapist, are aware of the latest developments and the potential utility of new treatment approaches. The critical analysis of these developments is desired.

The physiotherapist can aid the patient with PCa with simple procedure and the correct evaluation will help the professoional to take decisions. The assessment of the patient by the physiotherapist include an anamnesis, voiding diary, pad test, data collection of the urodynamic study and/or other complementary examinations, if any, physical examination and specific maneuvers to assess urine leakage (23, 29). In the interview, beyond identifying the main complaint and history of the patient with PCa, issues inherent in urination are of utmost importance to be addressed. The voiding diary is a useful tool because it allows the physiotherapist to objectively quantify the volume of urine loss, as well as the frequency of the urination. As the voiding diary is fully performed by the patient over a period of about two to three days, the type of the drink, volume voided, urgency severity, quantification of loss and its association to carry out some activity at the time, he is leading to observe his behavior voiding, generating his self-knowledge (23, 25-30).

Dorey *et al*, 2009 (23) have suggested to get information related to the patient about fluid intake, caffeine, diet and obesity, constipation, general fitness, lifting, chest problems and urinary tract infections. These autors described that taboo and embarrassment that affects help-seeking behaviour by people affected by incontinence may also affect the quality of self-reporting in research.

Urodynamic investigations can bring important information and involves the evaluation of the dynamic function of the lower urinary tract. The urodynamic study, an examination of the gold standard, evaluates the morphology, pressure (urethral, vesical and abdominal under static and dynamic conditions), physiology and hydrodynamic transport urine of the voiding mechanism, thus detailing the stages of filling and emptying as well as the sphincter behavior. Common urodynamic findings in post-RP patients are (a) internal sphincter deficiency and (b) bladder dysfunction (detrusor instability and decreased compliance) (23, 25-30).

The correct evaluation of the findings obtained in the interview and in the examinations would permit the professional to take simple decision as described by Dorey *et al,* 2009 (23).

A very important and unquestionable point is that pelvic floor muscle exercises are relevant to the treatment of ED and Ui in patients with PCa that will be submitted to RP (26, 27). One of the aims of the intervention of the physiotherapy is to re-train the muscles of the pelvis by improving the active retention strength of the striated muscles of the pelvic floor in order to overcome the insufficiency of the injured sphincters and improve the continence of men with PCa. This level includes the awareness of the pelvic floor musculature and the coordination of the contraction-relaxation process to improve the control and the quality of the muscle contraction. Exercises using a ball, in which, the patient is sat in it, may be worthwhile (7, 8, 23, 29, 30, 53).

The pelvic floor muscles play also an important role in sexual activity and contractions of the ischiocavernosus and bulbocavernosus muscles produce an increase in the intracavernous pressure and influence penile rigidity. The procedures of the physiotherapy, associated with an interdisciplinar team, including exercises for the muscles of the pelvic floor muscle only or associated with backmanometric biofeedback, electrotherapy, vaccum pumps can be used successfully in various patients with ED (8, 23, 25-30, 51-55, 56).

**Figure 2:**
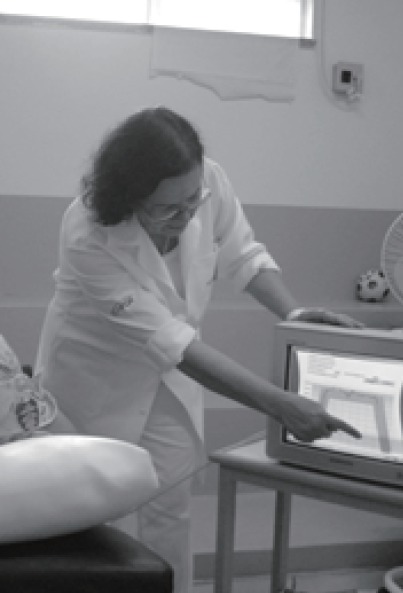
Physiotherapist and the use of the biofeedback in a patient

Biofeebakk is a helpful and suitable tool in learning to control contracting and relaxing of the pelvic floor muscles (23, 56). Figure 2 shows a physiotherapist that is teaching instructions about the biofeedback to the patient about the procedures involving the contractions and relaxations of the muscles of the pelvic floor that the patient can see in a monitor.

In addition, it is highly desired to consider that beneficial effects of pre- and postoperative pelvic floor interventions (RT or RP) using physiotherapy procedures, since both the duration and degree of UI after RP decrease in these case (23, 29, 53-55).

When a patient with PCa is referred to undertake physiotherapy procedures before the surgery or radiotherapy, it is possible to teach him about the perception of the muscles of the pelvic foor, facilitating the performance of exercises involving these muscles associated with an ideal breathing, just after the RP or RT (8, 25-30).

Besides the ED, another clinical conditions related to the sexual functions can appear in the patient submitted to a RP, as the loss of ejaculation and the decrease of the libido and orgasm (8, 34, 46, 48). The interventions related to the physiotherapy will contribute to aid the patient to live your sexuality and it is important to show to the patient that sexuality is not only genitality, but it goes beyond the limits of genital impulse and is characterized as a strong experience of human personality (8, 17, 34, 46, 48).

Several options of treatment are available to treat ED, as psychosexual counseling, medication, use of physiotherapy (exercises to the pelvic floor muscles, electrotherapy and external vacuum devices), intracavernous injection therapy, vascular surgery, and use of a penile prosthesis. The clinical interventions used in the physiotherapy provide noninvasive methods that are easy to perform, painless, and inexpensive (8, 23, 52, 53). Furthermore, authors (23) have recommended the contraction the pelvic floor muscles during sexual activity with the aaim to maintain or improve erectile strength.

Most physiotherapy treatments for ED focus also on pelvic floor muscles. It is relevant to consider also the arrangement of the muscles at the base of the penis, as well as the other local structures that, with the time without erection, can lead to veno-occlusive ED. ED can be a result of a sequence of penile morphologic alterations post-RP. The physiotherapist will guide the patient to do exercises for the muscles direct related to the pelvic floor and also to the muscles indirectly related with the pelvis, such as abdominal and gluteal muscles. When they are contracted an increase of the local blood flow to the pelvic region is verified. This process seems to lead to a release of NO to the penis, acting on endothelium vasodilation and dependent on the flow, increasing in oxygen supply to the penile tissue and keeping the erectile tissue healthy (23, 29, 53). Following this same point of view, the vaccum therapy could also provide oxygen supply generated by negative pressure that distends the corporal sinusoids and increases the blood inflow to the penis. The vaccum therapy could be combined with anothers therapies for ED, as pelvic floor muscles exercices (kinesiotherapy) and oral therapies (medications) (52).

The physiotherapist, from his assessment, can also help the patient with PCa in the presurgical period in which the exercises for the pelvic floor and for the respiration that will be performed in the post-surgical period can be learned early by the patient. Moreover, the knowledge and the perception of the muscles of the pelvic floor by the patient will be very important. As these muscles are located inside the pelvis, they are considered a continence muscle group giving structural support for the pelvic organs and the pelvic sphincters (urethra and anus, for exemple in men). Based on urethral continence maintained by muscles of the pelvic floor, the procedures of the physiotherapy of this muscle group can retake the control of the urinary continence or maximize it, also by nerve stimulation, according to the consensus, which can inhibit the detrusor muscle, increasing the quality of life of patients with PCa (23, 25-30).

On physical examination is evaluated the strength and the tone of the pelvic floor muscles through the anal sphincter, perineal sensation and bulb-cavernosum reflex. Maneuver effort, such as coughing, can evaluate the sphincter function, which can be performed with the patient standing, with the bladder full, and where he is asked to simulate cough. From this assessment is given the goal of treatment (23, 25-30).

**Figure 3:**
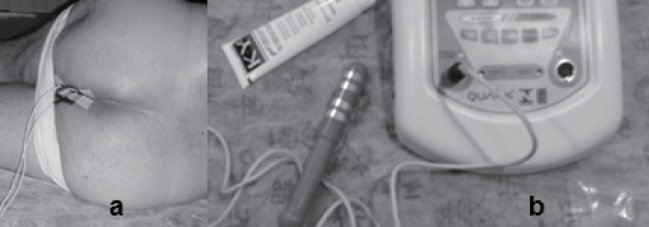
Patient after prostate cancer surgery undergoing electrotherapy

The perception of the muscles of the pelvic floor may be increased with the use of the electrotherapy and this technique might guide the patient to correct contraction of the muscles of the pelvic floor. Two types of electrodes can be used in the electrotherapy; internal (anal) and external electrodes (Figure 3a and 3b) (8, 23, 29, 54, 55, 57). In the case of functional electrical stimulation, which is an alternating current of low frequency, it generates muscle contractions and an increase of muscle function. In the pelvic floor muscles, electrode stimulation in the perineal body, the contraction is perceived by the patient and the physiotherapist with the apparent anal contraction. This contraction also acts by stimulating the sacral nerve roots, or specifically the pelvic and pudendal nerves, suppressing the (hyper) detrusor activity (23, 29).

In Figure 3a, a patient that is undergoing electrotherapy with external (surface) electrodes is shown. A correct frequency is choosen, following international studies and the intensity of electric current is selected considering the sensibility of the patient. The cable is connected to equipament in the Figure 3b, in which is also shown an anal electrode to internal stimulation.

Beneficial effects of pre- and postoperative pelvic floor re-education are clear, since both the duration and degree of UI after RP can be distinguishably decreased (7, 21, 54).

Concerning ED, when a man wakes up from a RP he will almost certainly have ED initially. If there is going to be a recovery of erectile function, it may take 18-24 months to occur. Approximately 30% of men will recover erectile function and medication (Viagra or Cialis) will usually boost this recovery. However, physiotherapy procedures could be another suitable option without contraindications. In addition, the patient that has learned about the exercises involving the muscles of the pelvic floor can start these exercises immediately just after the surgery or after the catheter removal (22, 23, 25-30, 32, 58).

Mechanical vacuum devices cause erection by creating a partial vacuum, which draws blood into the penis, engorging and expanding it. The physiotherapist will guide the patient to do exercises for the muscles direct related to the pelvic floor (23) and also to the muscles indirectly related with the pelvis, such as abdominal and gluteal muscles (27, 29, 53). When they are contracted an increase of the local blood flow to the pelvic region is verified. This process seems to lead to a release of NO to the penis, acting on endothelium vasodilation and dependent on the flow, increasing in oxygen supply to the penile tissue and keeping the erectile tissue healthy (23, 52, 58-60).

Another consideration is that loss of bone mineral density has been observed in PCa patients on ADT (61, 62). It has also been shown that ADT increases body weight and fat mass in these patients (63). Decreased levels of testosterone and body changes during ADT, may also influence mental health, fatigue and health-related quality of life (HRQOL) in PC patients (64, 65). PCa patients treated with ADT for 6-12 months, lean body mass (LBM) has been reported to decrease about 3% (63). This reduction may affect muscle strength markedly because muscle mass is the dominant tissue in LBM. Putting together these findings, whole body vibration exercises (WBV) performed in oscillating platform could be a good option to aid the patient with cancer. The vibrations generated in these platforms can be transmitted to body of the patient, and, it is suggested that, in appropriated conditions, these vibrations could improve walking function, muscle strength, bone mineral density, cardiovascular fitness and body balance. Moreover, the health-related quality of life is increased and the fall risk is decreased. Moreover, the level the testosterone in the plasma seems to increase due to the WBV. The mechanisms responsible for the WBV benefits are not fully understood, however it is hypothesized that these effects are probably related to direct and indirect actions. The direct effects would be related with the transmission of energy of the vibration, for example, to a muscle that would be stimulated. The indirect effects might to be associated with the neuroendocrine system. Whole body mechanical vibration on the muscle performance would be due to the induction of a myotatic reflex contraction referred as the tonic vibration reflex (66-68). The WBV could be also interesting to aid the PCa patient that has fatique, that is a undesirable clinical condition in this individual, as reported by Stone* et al,* 2002 (65) and Baumann *et al*, 2012 (20).

## DISCUSSION

Cancer is an important public problem and the World Health Organization (WHO) develops strategies towards the prevention, research, education and control of the cancer. Important medical developments and relevand scientific findings have permitted that people with cancer can survive with their disease and with the side effects of their disease and its treatment for longer (3, 68, 70) and this fact has reinforced the importance of the procedures used in the physiotherapy.

People are living longer with their cancers, which in many cases are treated as chronic disease, due to the early detection and advances in treatment options. Physiotherapy has responded to the improved outcomes and patient demand for quality of life improvements by instituting new treatments and education, such as informing about the possible importance of the sunlight in the prevention of the PCa (4, 5, 70) and the equal need to protect against the harmful effects of the ultraviolet radiation, or about the options of physiotherapy for rehabilitation and re-integration to normal life (7, 8, 9, 10). Moreover, the treatment of older cancer survivors must consider an accelerated functional decline; due, in part, to low physical activity levels and deficits in muscle and mobility and the kinesiotherapy is very relevant (4, 5, 13).

As Katz and Katz, 2008 (18) and Bernardo-Filho *et al*, 2009 (7), it is relevant that personal situations related with the possible treatments for PCa must be considered, as bladder irritation is common after RT, bowel complications might occur in the long-term and have high incidence during external beam RT, ED can be early in the surgery in comparison with RT, and penile shortening or fibrosis might occur after RP. Clinical conditions after the RP, such as pelvic pain, is common mainly in young men, UI will occur in the post operative period, erectile functioning might return slowly over years after the surgery. All these must be considered and must be explained to the patient and his family. The decline of the quality of the sexual activity can lead to a complicated pattern of change in quality of life and also negatively affect the psychosocial wellbeing of men and of the couple.

When the available procedures to minimize the clinical complications of the RP or of the RT are considered, it is highly relevant to emphasize that the decrease of the appearance of complications occurs in patients thar have undergone physiotherapy before the RP and the improvement of the symptoms is observed due to the procedures of the physiotherapy just after the RP (8, 18). During the final stages of cancer treatment, the palliative care becomes paramount and the participation of the physiotherapist is also desirable in the interdisciplinary team. The care with the patient with cancer will contribute to minimize the progression of secondary symptoms (7, 8, 31).

Dorey *et al*, 2009 (23) have reported that the the pelvic floor muscle exercises are effective to treat some men with ED. Moreover, they suggest that to obtain a benefit, pelvic floor muscle exercises should be properly taught and practised for at least 3 months. A maintenance programme may then be implemented for life. An important condition to be considered is that not all men with ED may be suitable for pelvic floor muscle training. Those men with severe arteriogenic and neurological causes may well not benefit. Pelvic floor muscle exercises could be considered as a first-line approach for men seeking resolution of ED without pharmacological and surgical interventions. Dorey *et al*, 2006 (29) suggest that men receiving other forms of therapy for ED could be advised to additionally practise pelvic floor muscle exercises. Moreover, although pelvic floor muscle exercises are more labour intensive than using a pharmacological agent, it is suggest that the men could be have a choice of treatment, considering that some men may prefer a more natural approach. Dorey *et al*, 2009 (23) described that instruction for the Pelvic Floor Muscle Training to men after prostate surgery (MAPS) with leaflet aims to support and re-inforce the anatomy teaching received during MAPS therapy appointments, as well as the exercise programme that had been set.

Zhu *et al*, 2012 (55) have evaluated the role of electrical stimulation (ES) in the recovery of postprostatectomy UI and a meta-analysis was carried out for all available randomized controlled trials comparing ES enhanced pelvic floor muscle training (PFMT) with PFMT alone for postprostatectomy UI. These authors, based on the available evidence, have demonstrated that ES enhanced PFMT did not improve the return to continence more than PFMT in men with postprostatectomy UI.

Khoder *et al*, 2011 (71), have evaluated 911 patients retrospectively with different grades of UI after RP for perioperative risk factors and effect of rehabilitation procedures. Ninety-six percent of patients suffered different grades of incontinence at beginning of hospitalization. Conservative therapy, including pelvic floor muscle training, anal electrical stimulation (AES) or combinations has been performed on all patients. Grade of UI after RP showed significant improvement after 3weeks rehabilitation period. These autors have concluded that preoperative counseling of patients should provide them with realistic expectations for UI after RP and motivate them to conservative therapy, as it reduces the duration and degree of UI.

Yamanishi *et al*, 2010 (57) have evaluated electrical stimulation combined with pelvic floor muscle training for UI after RP in a randomized controlled study. A total of 56 men with severe UI were randomized to an active treatment group or a sham group. All patients performed pelvic floor muscle training preoperatively and continued throughout the study. For active stimulation 50 Hz square waves of 67 μs pulse duration and a 5 seconds on, 5 seconds off duty cycle were applied for 15 minutes twice daily with an anal electrode. Sham stimulation was limited to 3 mA with a 2 seconds on, 13 seconds off duty cycle. The findings of these authors revealed that ES resulted in earlier recovery of continence in patients with UI after RP.

Wilson *et al,* 2005 (72) and Dorey *et al,* 2009 (23) has reported that drinks containing caffeine or alcohol may cause increased risk of urinary urgency, and men under treatment must be advised to moderate or avoid them.

Baumann *et al,* 2012 (20) have evaluated the evidence of randomized controlled studies which examined exercise during medical treatment and in the aftercare of a prostate cancer disease. They observed that current data suggest that UI, fitness, fatigue, body constitution, and also quality of life can be improved by clinical exercise in patients during and after prostate cancer.

In conclusion, the knowledge of the patient about his situation as well as the involvement of the family and partner must be strongly considered. Moreover, it is also important to explain and present all the possibilities involving the treatment of the PCa. In addition, it is highly desired thal all the modalities of procedures that are available to aid in the prevention of undesirable clinical conditions. Furthermore, it is suggested that is necessary to consider the techniques related to the physiotherapy before and after the treatment of choice to the PCa.
